# 3-(2-Amino­ethyl)-2-(4-fluoroanilino)­quinazolin-4(3*H*)-one

**DOI:** 10.1107/S1600536808035058

**Published:** 2008-10-31

**Authors:** Xu-Hong Yang, Ming-Hu Wu

**Affiliations:** a Faculty of Chemistry and Life Science, Xianning University, Xianning 437100, People’s Republic of China

## Abstract

In the title mol­ecule, C_16_H_15_FN_4_O, the dihedral angle between the fluoro-substituted benzene ring and the pyrimidinone ring is 52.34 (7)°, while the dihedral angle between the fused benzene ring and the pyrimidinone ring is 3.30 (6)°. An intra­molecular N—H⋯N hydrogen bond may, in part, influence the conformation of the mol­ecule. In the crystal structure, inter­molecular N—H⋯N hydrogen bonds and weak C—H⋯π(arene) inter­actions link pairs of mol­ecules into centrosymmetric dimers.

## Related literature

For the biological properties of quinazolinones and their derivatives, see: Armarego (1963 ▶); Witt & Bergman (2003 ▶). For details of our ongoing heterocyclic synthesis and drug discovery project, see: Yang *et al.* (2008 ▶).
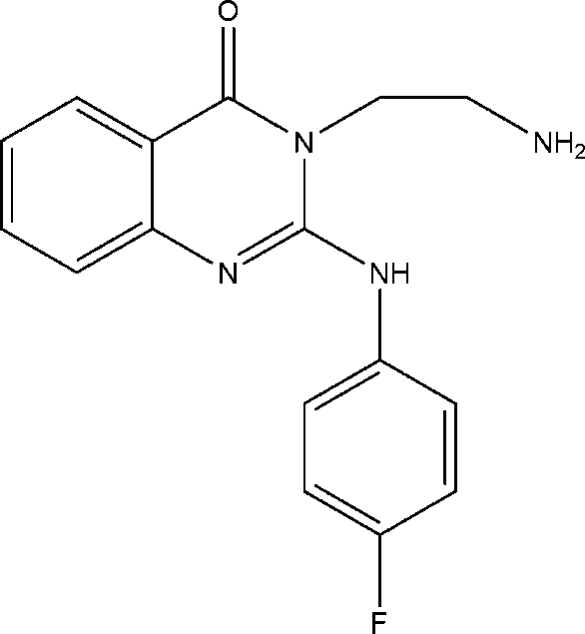


## Experimental

### 

#### Crystal data

C_16_H_15_FN_4_O
*M* *_r_* = 298.32
Triclinic, 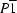

*a* = 8.2836 (8) Å
*b* = 9.3103 (10) Å
*c* = 9.4952 (10) Å
α = 89.36 (1)°
β = 80.537 (10)°
γ = 77.163 (10)°
*V* = 704.03 (13) Å^3^
*Z* = 2
Mo *K*α radiation
μ = 0.10 mm^−1^
*T* = 292 (2) K
0.20 × 0.10 × 0.10 mm

#### Data collection

Bruker SMART APEX CCD diffractometer
Absorption correction: multi-scan (*SADABS*; Bruker, 2001 ▶) *T*_min_ = 0.970, *T*_max_ = 0.990
4078 measured reflections
2726 independent reflections
2318 reflections with *I* > 2σ(*I*)
*R*_int_ = 0.012

#### Refinement

*R*[*F* ^2^ > 2σ(*F* ^2^)] = 0.038
*wR*(*F* ^2^) = 0.111
*S* = 1.05
2726 reflections
209 parameters
H atoms treated by a mixture of independent and constrained refinement
Δρ_max_ = 0.16 e Å^−3^
Δρ_min_ = −0.15 e Å^−3^

### 

Data collection: *SMART* (Bruker, 2007 ▶); cell refinement: *SAINT* (Bruker, 2007 ▶); data reduction: *SAINT*; program(s) used to solve structure: *SHELXS97* (Sheldrick, 2008 ▶); program(s) used to refine structure: *SHELXL97* (Sheldrick, 2008 ▶); molecular graphics: *PLATON* (Spek, 2003 ▶); software used to prepare material for publication: *SHELXTL* (Sheldrick, 2008 ▶).

## Supplementary Material

Crystal structure: contains datablocks global, I. DOI: 10.1107/S1600536808035058/lh2717sup1.cif

Structure factors: contains datablocks I. DOI: 10.1107/S1600536808035058/lh2717Isup2.hkl

Additional supplementary materials:  crystallographic information; 3D view; checkCIF report

## Figures and Tables

**Table 1:** Hydrogen-bond geometry (Å, °)

*D*—H⋯*A*	*D*—H	H⋯*A*	*D*⋯*A*	*D*—H⋯*A*
N1—H1⋯N4	0.908 (17)	1.925 (17)	2.8049 (17)	162.8 (14)
N4—H4*A*⋯N2^i^	0.889 (16)	2.323 (16)	3.1321 (18)	151.4 (13)
C3—H3⋯*Cg*^i^	0.93	2.77 (1)	3.4741 (15)	132 (1)

Symmetry code: (i) 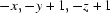. *Cg* is the centroid of atoms N2/C7/N3/C14/C13/C8.

**Fig. 1.**
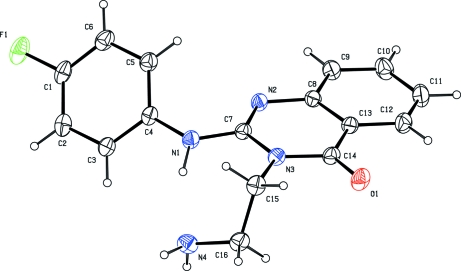
View of the title molecule with the atom-labeling scheme. Displacement ellipsoids are drawn at the 50% probability level.

**Fig. 2.**
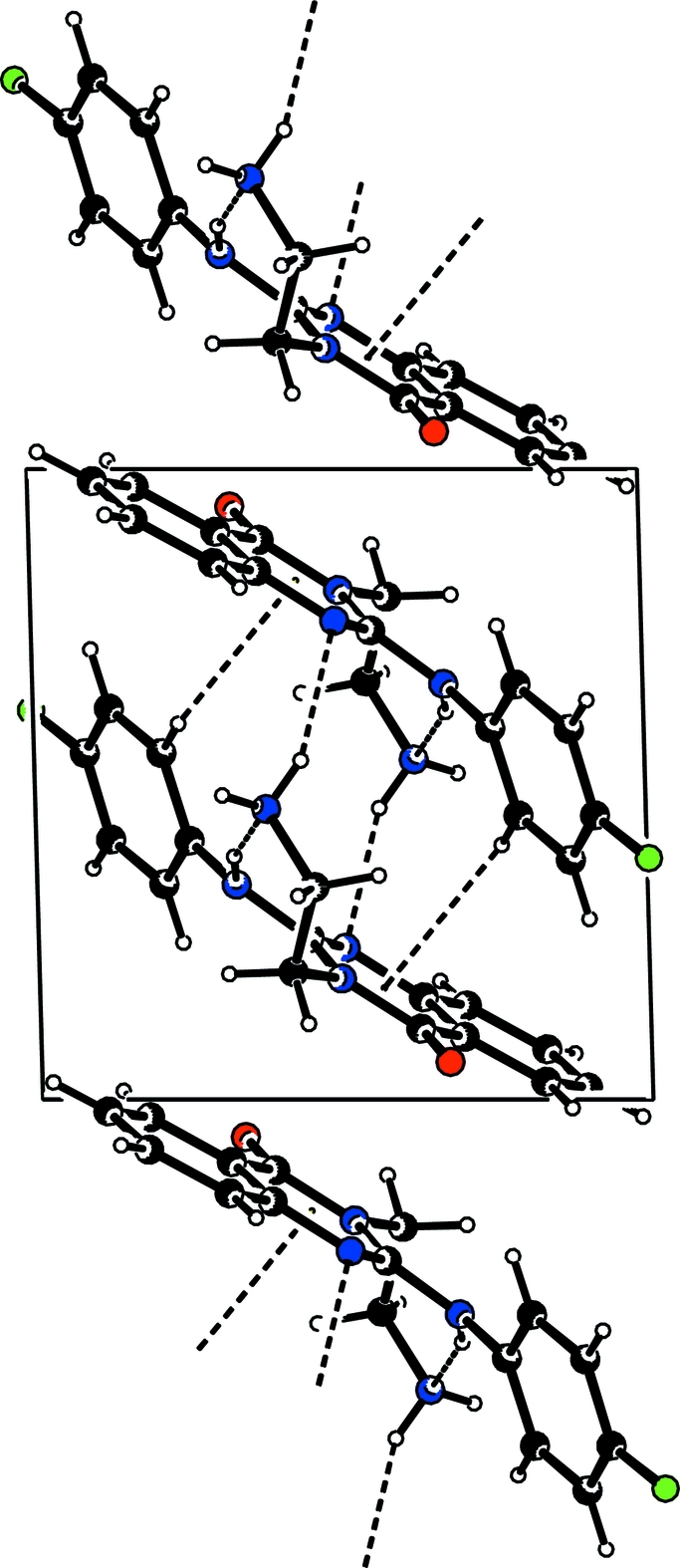
Part of the crystal structure of the title compound showing hydrogen bonds as dashed lines.

## supplementary crystallographic information

### Comment

Quinazolinones and their derivatives are now known to have a wide range of useful biological properties, such as hypnotic, sedative, analgesic, anti-convulsant, anti-tussive, anti-bacterial, anti-diabetic, anti-inflammatory and anti-tumor (Armarego, 1963; Witt & Bergman, 2003). In connection with our ongoing heterocyclic synthesis and drug discovery project (Yang *et al.*, 2008), we have focused our research on the synthesis of quinazolinones and pyrazolo pyrimidinones. Herein, the title compound was synthesized and its crystal structure was determined.

In the molecule (Fig. 1), the dihedral angle between the fluorophenyl and pyrimidinone ring is 52.34 (7)°, and the dihedral angle between the fused benzene ring and pyrimidinone ring is 3.30 (6)°. The torsion angles of N2–C7–N1–C4 and N3–C7–N1–C4 are -4.7 (2) and 176.42 (11)°, respectively.

An intramolecular N-H···N hydrogen bond may, in part, influence the conformation of the molecule. In the crystal structure, intermolecular N-H···N hydrogen bonds and weak C–H···π(arene) interactions link pairs of molecules into centrosymmetric dimers (see Table 1 and Fig. 2).

### Experimental

To a solution of 2-ethoxycarbonyliminophosphorane (1.27 g, 3 mmol) in 10 ml anhydrous THF, 4-chlorophenylisocyanate (0.46 g, 3 mmol) was added dropwise at room temperature. The reaction mixture was left unstirred for 6 h at 273–278 K, whereafter the above resulting solution was added dropwise to a solution of ethylenediamine (0.18 g, 3 mmol) in 5 ml anhydrous THF. After that, the reaction mixture was stirred overnight, the reaction mixture was cooled and the reaction product was recrystallized from CH_3_OH—CH_2_Cl_2_ to give colorless crystals of the title compound in yield 85%, which were suitable for X-ray analysis.

### Refinement

H atoms bonded to C atoms were placed in calculated positions (C—H = 0.93–0.97 Å) and included in the riding model approximation. The positional parameters of H atoms bonded to N atoms were refined independently. For all H atoms *U*_iso_ (H) = 1.2*U*_iso_ (C,N).

### Crystal data

**Table:**

C_16_H_15_FN_4_O	*Z* = 2
*M**_r_* = 298.32	*F*(000) = 312
Triclinic, *P*1	*D*_x_ = 1.407 Mg m^−^^3^
Hall symbol: -P 1	Mo *K*α radiation, λ = 0.71073 Å
*a* = 8.2836 (8) Å	Cell parameters from 2276 reflections
*b* = 9.3103 (10) Å	θ = 2.2–28.9°
*c* = 9.4952 (10) Å	µ = 0.10 mm^−^^1^
α = 89.36 (1)°	*T* = 292 K
β = 80.537 (10)°	Block, colourless
γ = 77.163 (10)°	0.20 × 0.10 × 0.10 mm
*V* = 704.03 (13) Å^3^	

### Data collection

**Table:**

Bruker SMART APEX CCD diffractometer	2726 independent reflections
Radiation source: fine-focus sealed tube	2318 reflections with *I* > 2σ(*I*)
graphite	*R*_int_ = 0.012
ω scans	θ_max_ = 26.0°, θ_min_ = 2.2°
Absorption correction: multi-scan (SADABS; Bruker, 2001)	*h* = −9→10
*T*_min_ = 0.970, *T*_max_ = 0.990	*k* = −11→11
4078 measured reflections	*l* = −11→11

### Refinement

**Table:**

Refinement on *F*^2^	Secondary atom site location: difference Fourier map
Least-squares matrix: full	Hydrogen site location: inferred from neighbouring sites
*R*[*F*^2^ > 2σ(*F*^2^)] = 0.038	H atoms treated by a mixture of independent and constrained refinement
*wR*(*F*^2^) = 0.111	*w* = 1/[σ^2^(*F*_o_^2^) + (0.0572*P*)^2^ + 0.1074*P*] where *P* = (*F*_o_^2^ + 2*F*_c_^2^)/3
*S* = 1.05	(Δ/σ)_max_ = 0.001
2726 reflections	Δρ_max_ = 0.16 e Å^−^^3^
209 parameters	Δρ_min_ = −0.15 e Å^−^^3^
0 restraints	Extinction correction: SHELXL97 (Sheldrick, 2008), Fc^*^=kFc[1+0.001xFc^2^λ^3^/sin(2θ)]^-1/4^
Primary atom site location: structure-invariant direct methods	Extinction coefficient: 0.033 (5)

### Special details

**Table:**

Geometry. All e.s.d.'s (except the e.s.d. in the dihedral angle between two l.s. planes) are estimated using the full covariance matrix. The cell e.s.d.'s are taken into account individually in the estimation of e.s.d.'s in distances, angles and torsion angles; correlations between e.s.d.'s in cell parameters are only used when they are defined by crystal symmetry. An approximate (isotropic) treatment of cell e.s.d.'s is used for estimating e.s.d.'s involving l.s. planes.
Refinement. Refinement of *F*^2^ against ALL reflections. The weighted *R*-factor *wR* and goodness of fit *S* are based on *F*^2^, conventional *R*-factors *R* are based on *F*, with *F* set to zero for negative *F*^2^. The threshold expression of *F*^2^ > σ(*F*^2^) is used only for calculating *R*-factors(gt) *etc*. and is not relevant to the choice of reflections for refinement. *R*-factors based on *F*^2^ are statistically about twice as large as those based on *F*, and *R*- factors based on ALL data will be even larger.

### Fractional atomic coordinates and isotropic or equivalent isotropic displacement parameters (Å^2^)

**Table:**

	*x*	*y*	*z*	*U*_iso_*/*U*_eq_	
C1	0.26560 (18)	0.91647 (15)	0.45066 (18)	0.0596 (4)	
C2	0.1522 (2)	0.87525 (15)	0.37986 (16)	0.0589 (4)	
H2	0.1451	0.9031	0.2863	0.071*	
C3	0.04783 (17)	0.79107 (14)	0.45047 (14)	0.0503 (3)	
H3	−0.0308	0.7624	0.4040	0.060*	
C4	0.05872 (15)	0.74874 (13)	0.58961 (14)	0.0449 (3)	
C5	0.17665 (17)	0.79171 (15)	0.65820 (16)	0.0536 (3)	
H5	0.1860	0.7632	0.7512	0.064*	
C6	0.28049 (18)	0.87722 (16)	0.58782 (18)	0.0604 (4)	
H6	0.3590	0.9074	0.6333	0.073*	
C7	−0.02621 (15)	0.55612 (14)	0.74400 (13)	0.0424 (3)	
C8	0.15458 (16)	0.37171 (14)	0.83809 (13)	0.0444 (3)	
C9	0.31903 (18)	0.30166 (17)	0.85105 (16)	0.0573 (4)	
H9	0.4075	0.3440	0.8121	0.069*	
C10	0.3511 (2)	0.17169 (18)	0.92037 (17)	0.0670 (4)	
H10	0.4613	0.1260	0.9272	0.080*	
C11	0.2208 (2)	0.10669 (18)	0.98092 (18)	0.0690 (4)	
H11	0.2438	0.0177	1.0271	0.083*	
C12	0.0591 (2)	0.17479 (17)	0.97182 (16)	0.0587 (4)	
H12	−0.0284	0.1326	1.0136	0.070*	
C13	0.02374 (16)	0.30663 (14)	0.90063 (13)	0.0459 (3)	
C14	−0.14830 (17)	0.37725 (15)	0.88772 (14)	0.0479 (3)	
C15	−0.33989 (16)	0.58408 (16)	0.79762 (15)	0.0523 (3)	
H15A	−0.3462	0.6893	0.8010	0.063*	
H15B	−0.4153	0.5613	0.8799	0.063*	
C16	−0.39955 (16)	0.54589 (16)	0.66308 (16)	0.0555 (4)	
H16A	−0.3651	0.4403	0.6447	0.067*	
H16B	−0.5212	0.5731	0.6772	0.067*	
F1	0.36536 (13)	1.00298 (12)	0.38271 (13)	0.0901 (4)	
N1	−0.06114 (14)	0.67357 (13)	0.65917 (13)	0.0496 (3)	
H1	−0.151 (2)	0.6763 (17)	0.6150 (16)	0.060*	
N2	0.12716 (13)	0.49763 (12)	0.75954 (11)	0.0459 (3)	
H4A	−0.2947 (19)	0.5633 (17)	0.4621 (17)	0.055*	
H4B	−0.411 (2)	0.6934 (17)	0.5178 (15)	0.055*	
N3	−0.16619 (13)	0.50507 (12)	0.80814 (11)	0.0448 (3)	
N4	−0.33145 (16)	0.62199 (16)	0.53930 (14)	0.0590 (3)	
O1	−0.27139 (13)	0.33072 (12)	0.94041 (12)	0.0654 (3)	

### Atomic displacement parameters (Å^2^)

**Table:**

	*U*^11^	*U*^22^	*U*^33^	*U*^12^	*U*^13^	*U*^23^
C1	0.0477 (8)	0.0456 (7)	0.0809 (10)	−0.0143 (6)	0.0073 (7)	0.0107 (7)
C2	0.0648 (9)	0.0473 (7)	0.0579 (8)	−0.0098 (7)	0.0053 (7)	0.0068 (6)
C3	0.0511 (8)	0.0419 (7)	0.0559 (8)	−0.0093 (6)	−0.0050 (6)	0.0015 (6)
C4	0.0364 (6)	0.0387 (6)	0.0566 (7)	−0.0069 (5)	−0.0015 (5)	0.0050 (5)
C5	0.0434 (7)	0.0536 (8)	0.0647 (8)	−0.0136 (6)	−0.0081 (6)	0.0092 (6)
C6	0.0413 (7)	0.0536 (8)	0.0880 (11)	−0.0148 (6)	−0.0097 (7)	0.0079 (7)
C7	0.0381 (7)	0.0470 (7)	0.0433 (6)	−0.0146 (5)	−0.0034 (5)	0.0022 (5)
C8	0.0434 (7)	0.0514 (7)	0.0409 (6)	−0.0159 (6)	−0.0071 (5)	0.0053 (5)
C9	0.0435 (8)	0.0697 (9)	0.0620 (8)	−0.0185 (7)	−0.0115 (6)	0.0179 (7)
C10	0.0518 (9)	0.0761 (10)	0.0731 (10)	−0.0097 (8)	−0.0183 (7)	0.0242 (8)
C11	0.0698 (10)	0.0655 (10)	0.0738 (10)	−0.0170 (8)	−0.0177 (8)	0.0287 (8)
C12	0.0584 (9)	0.0619 (9)	0.0592 (8)	−0.0235 (7)	−0.0063 (7)	0.0159 (7)
C13	0.0462 (7)	0.0517 (7)	0.0421 (6)	−0.0177 (6)	−0.0050 (5)	0.0039 (5)
C14	0.0447 (7)	0.0547 (7)	0.0460 (7)	−0.0201 (6)	−0.0007 (5)	0.0026 (6)
C15	0.0348 (7)	0.0582 (8)	0.0595 (8)	−0.0086 (6)	0.0028 (6)	0.0021 (6)
C16	0.0334 (7)	0.0580 (8)	0.0755 (9)	−0.0106 (6)	−0.0095 (6)	0.0043 (7)
F1	0.0763 (7)	0.0820 (7)	0.1149 (9)	−0.0407 (6)	0.0059 (6)	0.0289 (6)
N1	0.0379 (6)	0.0532 (7)	0.0607 (7)	−0.0154 (5)	−0.0099 (5)	0.0125 (5)
N2	0.0382 (6)	0.0514 (6)	0.0498 (6)	−0.0157 (5)	−0.0054 (4)	0.0094 (5)
N3	0.0353 (6)	0.0517 (6)	0.0476 (6)	−0.0142 (5)	−0.0013 (4)	0.0029 (5)
N4	0.0429 (7)	0.0710 (8)	0.0621 (8)	−0.0101 (6)	−0.0096 (6)	0.0019 (6)
O1	0.0476 (6)	0.0723 (7)	0.0788 (7)	−0.0275 (5)	0.0011 (5)	0.0166 (5)

### Geometric parameters (Å, °)

**Table:**

C1—C2	1.362 (2)	C10—C11	1.392 (2)
C1—F1	1.3626 (16)	C10—H10	0.9300
C1—C6	1.366 (2)	C11—C12	1.365 (2)
C2—C3	1.3819 (19)	C11—H11	0.9300
C2—H2	0.9300	C12—C13	1.3914 (19)
C3—C4	1.3859 (19)	C12—H12	0.9300
C3—H3	0.9300	C13—C14	1.4551 (19)
C4—C5	1.388 (2)	C14—O1	1.2254 (15)
C4—N1	1.4123 (16)	C14—N3	1.3955 (17)
C5—C6	1.3870 (19)	C15—N3	1.4821 (16)
C5—H5	0.9300	C15—C16	1.516 (2)
C6—H6	0.9300	C15—H15A	0.9700
C7—N2	1.2980 (16)	C15—H15B	0.9700
C7—N1	1.3581 (17)	C16—N4	1.464 (2)
C7—N3	1.3956 (15)	C16—H16A	0.9700
C8—N2	1.3785 (16)	C16—H16B	0.9700
C8—C9	1.3981 (19)	N1—H1	0.908 (17)
C8—C13	1.4023 (18)	N4—H4A	0.889 (16)
C9—C10	1.366 (2)	N4—H4B	0.872 (16)
C9—H9	0.9300		
			
C2—C1—F1	118.49 (14)	C10—C11—H11	120.3
C2—C1—C6	122.61 (13)	C11—C12—C13	120.67 (14)
F1—C1—C6	118.88 (15)	C11—C12—H12	119.7
C1—C2—C3	118.37 (14)	C13—C12—H12	119.7
C1—C2—H2	120.8	C12—C13—C8	120.13 (13)
C3—C2—H2	120.8	C12—C13—C14	120.69 (12)
C2—C3—C4	120.93 (13)	C8—C13—C14	119.18 (12)
C2—C3—H3	119.5	O1—C14—N3	120.70 (12)
C4—C3—H3	119.5	O1—C14—C13	124.22 (13)
C3—C4—C5	119.19 (12)	N3—C14—C13	115.08 (11)
C3—C4—N1	117.79 (12)	N3—C15—C16	113.90 (11)
C5—C4—N1	122.81 (12)	N3—C15—H15A	108.8
C6—C5—C4	119.92 (14)	C16—C15—H15A	108.8
C6—C5—H5	120.0	N3—C15—H15B	108.8
C4—C5—H5	120.0	C16—C15—H15B	108.8
C1—C6—C5	118.98 (14)	H15A—C15—H15B	107.7
C1—C6—H6	120.5	N4—C16—C15	111.68 (12)
C5—C6—H6	120.5	N4—C16—H16A	109.3
N2—C7—N1	120.96 (11)	C15—C16—H16A	109.3
N2—C7—N3	124.41 (11)	N4—C16—H16B	109.3
N1—C7—N3	114.62 (11)	C15—C16—H16B	109.3
N2—C8—C9	119.28 (12)	H16A—C16—H16B	107.9
N2—C8—C13	122.38 (12)	C7—N1—C4	124.69 (11)
C9—C8—C13	118.27 (12)	C7—N1—H1	114.5 (10)
C10—C9—C8	120.65 (14)	C4—N1—H1	115.2 (10)
C10—C9—H9	119.7	C7—N2—C8	117.77 (11)
C8—C9—H9	119.7	C14—N3—C7	121.02 (11)
C9—C10—C11	120.84 (14)	C14—N3—C15	116.82 (11)
C9—C10—H10	119.6	C7—N3—C15	122.16 (11)
C11—C10—H10	119.6	C16—N4—H4A	112.6 (10)
C12—C11—C10	119.42 (14)	C16—N4—H4B	109.1 (10)
C12—C11—H11	120.3	H4A—N4—H4B	106.8 (14)
			
F1—C1—C2—C3	−178.16 (12)	C8—C13—C14—O1	−178.72 (13)
C6—C1—C2—C3	0.4 (2)	C12—C13—C14—N3	−177.09 (12)
C1—C2—C3—C4	−0.4 (2)	C8—C13—C14—N3	1.78 (18)
C2—C3—C4—C5	−0.1 (2)	N3—C15—C16—N4	−78.30 (15)
C2—C3—C4—N1	174.74 (12)	N2—C7—N1—C4	−4.7 (2)
C3—C4—C5—C6	0.7 (2)	N3—C7—N1—C4	176.42 (11)
N1—C4—C5—C6	−173.93 (12)	C3—C4—N1—C7	138.77 (13)
C2—C1—C6—C5	0.2 (2)	C5—C4—N1—C7	−46.58 (19)
F1—C1—C6—C5	178.68 (12)	N1—C7—N2—C8	−174.97 (11)
C4—C5—C6—C1	−0.7 (2)	N3—C7—N2—C8	3.84 (19)
N2—C8—C9—C10	−175.78 (14)	C9—C8—N2—C7	176.85 (12)
C13—C8—C9—C10	1.3 (2)	C13—C8—N2—C7	−0.13 (19)
C8—C9—C10—C11	−0.6 (3)	O1—C14—N3—C7	−177.93 (12)
C9—C10—C11—C12	−0.6 (3)	C13—C14—N3—C7	1.58 (17)
C10—C11—C12—C13	1.2 (3)	O1—C14—N3—C15	3.07 (19)
C11—C12—C13—C8	−0.5 (2)	C13—C14—N3—C15	−177.42 (11)
C11—C12—C13—C14	178.40 (13)	N2—C7—N3—C14	−4.69 (19)
N2—C8—C13—C12	176.23 (12)	N1—C7—N3—C14	174.18 (11)
C9—C8—C13—C12	−0.8 (2)	N2—C7—N3—C15	174.25 (12)
N2—C8—C13—C14	−2.65 (19)	N1—C7—N3—C15	−6.87 (17)
C9—C8—C13—C14	−179.65 (12)	C16—C15—N3—C14	−96.27 (14)
C12—C13—C14—O1	2.4 (2)	C16—C15—N3—C7	84.75 (15)

### Hydrogen-bond geometry (Å, °)

**Table:**

*D*—H···*A*	*D*—H	H···*A*	*D*···*A*	*D*—H···*A*
N1—H1···N4	0.908 (17)	1.925 (17)	2.8049 (17)	162.8 (14)
N4—H4A···N2^i^	0.889 (16)	2.323 (16)	3.1321 (18)	151.4 (13)
C3—H3···Cg^i^	0.93	2.77 (1)	3.4741 (15)	132 (1)

Symmetry codes: (i) −*x*, −*y*+1, −*z*+1.
